# SPDEv3.0: A Swiss Army knife for plant genomics and breeding analysis

**DOI:** 10.1093/plphys/kiaf545

**Published:** 2025-10-27

**Authors:** Neeta Lohani

**Affiliations:** Plant Physiology, American Society of Plant Biologists; Department of Biotechnology, Thapar Institute for Engineering and Technology, Patiala, Punjab 147004, INDIA

High-throughput sequencing technologies have transformed plant biology research over recent decades. Whole genome sequencing, which once required years of work and substantial resources, now takes days at a fraction of the cost. This technological advancement has generated massive datasets across diverse plant species, enabling researchers to analyze genetic variation, evolutionary processes, and complex traits at unprecedented scales (Xie et al. 2024). The impact extends from fundamental biology to applied breeding programs, where genomic data now inform selection decisions and accelerate variety development. However, this data explosion has exposed a bottleneck: while generating biological datasets has become routine, analyzing them requires navigating multiple software tools, each with different interfaces, file formats, and dependencies. A typical comparative genomics workflow might involve BLAST for sequence alignment, custom scripts for data parsing, MCScanX for collinearity analysis (Wang et al. 2012), and R for visualization. Each transition between tools introduces potential errors and compatibility issues. While computational expertise, whether developed in-house or through collaboration, can address these challenges, the fragmentation of analytical workflows increases time, cost, and the potential for errors. Consequently, streamlining these processes could significantly improve research efficiency (Berger and Yu 2023).

In this issue of *Plant Physiology*, Xu et al. (2025) present SPDEv3.0, an integrated bioinformatics platform designed to streamline complex genomic analyses. The platform consolidates over 130 functions across 7 core modules—Gene, Genes, Alignment, Genome, Breeding, File Edit, and Visualization—into a unified graphical interface (Fig. 1A). SPDEv3.0 handles diverse tasks, including gene family identification, gene structure annotation, sequence alignment, collinearity analysis (genome comparison), phylogenetic tree construction, and promoter element analysis, while incorporating automated parameter optimization and batch processing capabilities. This integration reduces the need to switch between multiple software environments, enabling researchers to move from raw sequence data to publication-ready results within a single platform.

**Figure 1.**
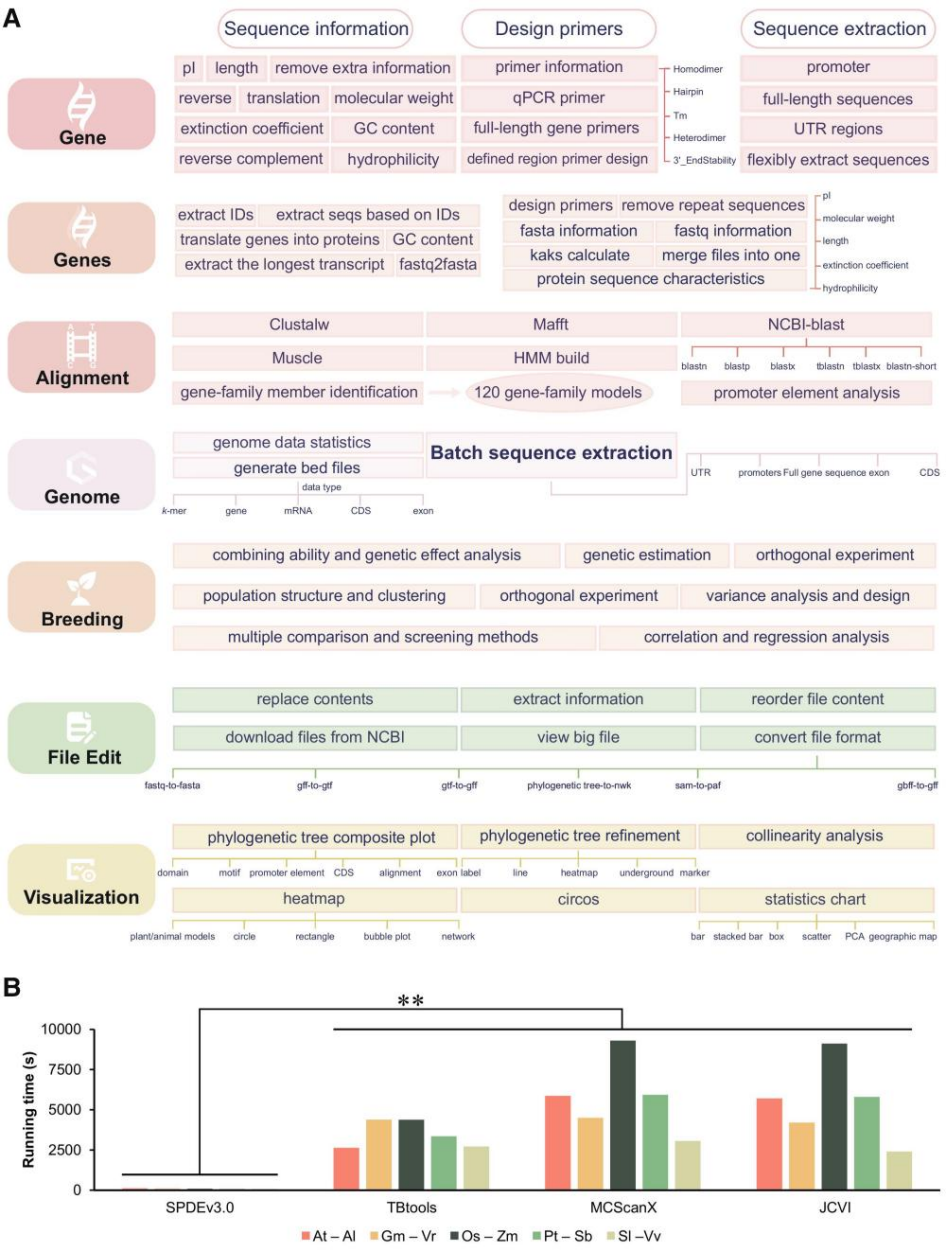
SPDEv3.0 integrates 130+ functions across 7 modules with exceptional speed. **A)** The platform organizes bioinformatics workflows into 7 interconnected modules: Gene (sequence analysis and primer design), Genes (batch processing), Alignment (gene family identification and multiple alignment tools), Genome (feature extraction and statistics), Breeding (40 classical breeding methods), File Edit (format conversion and data management), and Visualization (publication-ready graphics including phylogenetic trees, Circos plots, and heatmaps). **B)** Performance benchmark comparing collinearity analysis speed across different platforms. SPDEv3.0 completed synteny block detection in under 2 min for all 5 genome pairs tested, significantly outperforming TBtools, MCScanX, and JCVI (**, *P* ≤ 0.01). Genome pairs tested: *Arabidopsis thaliana–A. lyrata* (At–Al, 115 to 199 Mb), *Glycine max–Vigna radiata* (Gm–Vr, 945 to 448 Mb), *Oryza sativa–Zea mays* (Os–Zm, 373 Mb–2.05 Gb), *Populus trichocarpa–Salix babylonica* (Pt–Sb, 378 to 369 Mb), and *Solanum lycopersicum–Vitis vinifera* (Sl–Vv, 804 to 478 Mb).

SPDEv3.0's primary innovation lies in automating analytical workflows that traditionally require manual intervention at multiple steps. Consider collinearity analysis, a fundamental task in comparative genomics and polyploid crop research. Conventional approaches using tools like MCScanX require researchers to manually extract coding sequences, translate them to proteins, perform alignments, identify syntenic blocks, and generate visualizations (Wang et al. 2012, 2024). Each step requires specific file formats and separate software packages. SPDEv3.0 consolidates this pipeline into a single automated workflow. Users provide genome FASTA files and GFF annotations, and the platform executes all intermediate steps automatically. The authors demonstrate that collinearity analysis between *Arabidopsis thaliana* and *A. halleri* completed in under 2 minutes, substantially reducing the time investment required for such analyses (Fig. 1B).

This automation extends across the platform's architecture. The Gene module handles sequence operations, including promoter and UTR extraction with flexible region definitions, as well as primer design for qPCR and gene cloning. The Genes module scales these capabilities for high-throughput applications through batch processing, scaling these capabilities for high-throughput applications. The Alignment module integrates multiple sequence alignment tools—BLAST, Diamond, ClustalW, MAFFT, and MUSCLE—behind a single graphical interface, eliminating multiple tool integration or command-line requirements. The Gene Family module employs Hidden Markov Models for protein domain identification, supporting both preloaded models for 120 well-characterized plant gene families and custom model construction. Each module's output can serve as input for downstream analyses, creating integrated pipelines without manual data reformatting.

Beyond genomic analysis, SPDEv3.0 addresses an important gap in plant breeding research by integrating over 40 classical statistical methods directly into the platform. Plant breeding programs routinely employ quantitative genetics approaches, including heritability estimation, variance component analysis, combining ability assessment, and orthogonal experimental designs. These analyses traditionally require specialized statistical software like SAS or R, which, while powerful, demand programming proficiency and often necessitate collaboration with statisticians or investment in training. SPDEv3.0's Breeding module implements these methods with automated computation following user data input, supporting multi-environment trials, genetic correlation analysis, and population structure assessment. This integration allows researchers to conduct both genomic and breeding analyses within a single environment, potentially reducing the time and coordination required for translational research connecting molecular findings to phenotypic outcomes.

Furthermore, the platform's visualization capabilities demonstrate its integrated design philosophy. Rather than treating visualization as a separate downstream step requiring data export, the Visualization module provides over 30 plot types directly coupled to upstream analyses. Genomic feature maps display annotation structures from GFF files. Domain architecture diagrams render protein structure outputs from gene family analyses. Collinearity plots are generated immediately following synteny detection. Statistical charts, including heatmaps, PCA plots, and violin plots, produce figures without requiring data export or format conversion. Users can customize visual parameters such as colors, layouts, and annotation details through the graphical interface. By generating figures directly from analyses, the platform avoids the usual step of reformatting outputs for external graphing tools, thereby accelerating the transition from results to insights.

An important consideration for integrated platforms is whether consolidation affects computational performance, particularly when handling large genomes. The authors address this through systematic benchmarking across genome sizes spanning 3 orders of magnitude, from *A. thaliana* (135 mb) to *Allium cepa* (14 Gb). The platform employs memory-efficient architecture through the *pyfaidx* library, enabling indexed, region-specific genome access rather than loading entire genomes into memory (Shirley et al. 2015). For the *A. cepa* genome, GFF annotation visualization completed in under 7 minutes on standard desktop hardware. Performance comparisons showed SPDEv3.0 achieving comparable or faster execution times relative to established tools like MCScanX and TBtools (Chen et al. 2020, 2023) while maintaining accuracy. The authors validated analytical precision using benchmark datasets for gene family identification, achieving high precision and recall across families with diverse domain architectures. These results indicate that integration and accessibility need not compromise computational efficiency or analytical accuracy.

SPDEv3.0's integrated design offers practical advantages across multiple research areas. In comparative genomics, researchers studying polyploid crop evolution can trace homoeologous relationships and chromosomal rearrangements using automated collinearity workflows. In molecular breeding, programs can integrate genomic selection with classical quantitative genetics approaches without switching platforms. In functional genomics, investigations can flow continuously from gene family identification through phylogenetic analysis to visualization, all within the same environment. By consolidating these diverse analytical tasks, the platform reduces both the time invested in managing different software packages and the technical errors that often arise when transferring data between incompatible tools.

Additionally, the platform's modular architecture is designed in such a way that it allows for continued expansion. The authors outline plans to integrate machine learning capabilities, including supervised models for trait prediction and graph neural networks for biological network inference. These additions would extend SPDEv3.0's functionality beyond current descriptive analyses toward predictive modeling. However, the platform does have some limitations. It works best with complete genome datasets, but certain specialized analyses, such as those with incomplete datasets, small sample sizes, or large-scale phenotyping data, may need additional customization. The authors plan to address these limitations by expanding data compatibility and analytical capabilities in future versions.

More broadly, SPDEv3.0 represents an important principle in computational biology: that reducing technical barriers in data analysis can improve research efficiency across the community. As genomic data generation continues to outpace the availability of specialized computational expertise, tools that streamline analytical workflows become increasingly valuable. By demonstrating that sophisticated bioinformatic analysis can be made more accessible without sacrificing analytical capability or computational performance, SPDEv3.0 offers a practical model for integrated platforms in plant science. For research groups seeking to leverage genomic data more efficiently, whether through reduced analysis time, decreased need for specialized programming, or improved reproducibility through standardized workflows, this platform represents a useful addition to the computational toolkit.

## Data Availability

No new data were generated or analyzed in support of this article.

## References

[kiaf545-B1] Berger B, Yu YW. Navigating bottlenecks and trade-offs in genomic data analysis. Nat Rev Genet. 2023:24(4):235–250. 10.1038/s41576-022-00551-z 36476810 PMC10204111

[kiaf545-B2] Chen C, Chen H, Zhang Y, Thomas HR, Frank MH, He Y, Xia R. TBtools: an integrative toolkit developed for interactive analyses of big biological data. Mol Plant. 2020:13(8):1194–1202. 10.1016/j.molp.2020.06.009 32585190

[kiaf545-B3] Chen C, Wu Y, Li J, Wang X, Zeng Z, Xu J, Liu Y, Feng J, Chen H, He Y, et al TBtools-II: a “one for all, all for one” bioinformatics platform for biological big-data mining. Mol Plant. 2023:16(11):1733–1742. 10.1016/j.molp.2023.09.010 37740491

[kiaf545-B4] Shirley MD, Ma Z, Pedersen BS, Wheelan SJ. Efficient” pythonic” access to FASTA files using pyfaidx. 2015, PeerJ Preprints. 10.7287/peerj.preprints.970v1

[kiaf545-B5] Wang Y, Tang H, DeBarry JD, Tan X, Li J, Wang X, Lee T-h, Jin H, Marler B, Guo H, et al MCScanX: a toolkit for detection and evolutionary analysis of gene synteny and collinearity. Nucleic Acids Res. 2012:40(7):e49–e49. 10.1093/nar/gkr1293 22217600 PMC3326336

[kiaf545-B6] Wang Y, Tang H, Wang X, Sun Y, Joseph PV, Paterson AH. Detection of colinear blocks and synteny and evolutionary analyses based on utilization of MCScanX. Nat Protoc. 2024:19(7):2206–2229. 10.1038/s41596-024-00968-2 38491145

[kiaf545-B7] Xie L, Gong X, Yang K, Huang Y, Zhang S, Shen L, Sun Y, Wu D, Ye C, Zhu Q-H, et al Technology-enabled great leap in deciphering plant genomes. Nat Plants. 2024:10(4):551–566. 10.1038/s41477-024-01655-6 38509222

[kiaf545-B8] Xu D, Jin K, Zhang Q, Zhao X, Li Y, Wu T, Wang X, Yuan Y, An Z, Deng Z, et al SPDEv3.0: a multidisciplinary integrated data analysis platform. Plant Physiol. 2025: 199(3):kiaf537. 10.1093/plphys/kiaf537 41129697 PMC12596362

